# Clinical-pathological and molecular characterization of long-term survivors with advanced non-small cell lung cancer

**DOI:** 10.20892/j.issn.2095-3941.2019.0363

**Published:** 2020-05-15

**Authors:** Juan Moreno-Rubio, Santiago Ponce, Rosa Álvarez, María Eugenia Olmedo, Sandra Falagan, Xabier Mielgo, Fátima Navarro, Patricia Cruz, Luis Cabezón-Gutiérrez, Carlos Aguado, Gonzalo Colmenarejo, Marta Muñoz-Fernández de Leglaria, Ana Belén Enguita, María Cebollero, Amparo Benito, Isabel Alemany, Carolina del Castillo, Ricardo Ramos, Ana Ramírez de Molina, Enrique Casado, Maria Sereno

**Affiliations:** ^1^IMDEA Food Institute, CEI UAM + CSIC, Madrid 28702, Spain; ^2^12 de Octubre University Hospital, Madrid 28041, Spain; ^3^Gregorio Marañón University Hospital, Madrid 28009, Spain; ^4^Ramon y Cajal University Hospital, Madrid 28034, Spain; ^5^Infanta Sofía University Hospital, San Sebastián De Los Reyes, Madrid 28702, Spain; ^6^Fundación Alcorcon University Hospital, Alcorcon, Madrid 28922, Spain; ^7^Príncipe de Asturias University Hospital, Alcalá De Henares, Madrid 28805, Spain; ^8^La Paz University Hospital, Madrid 28046, Spain; ^9^University Hospital of Torrejón, Torrejón De Ardoz, Madrid 28850, Spain; ^10^Clinico San Carlos University Hospital, Madrid 28040, Spain; ^11^Parque Científico de Madrid Foundation, Madrid 28049, Spain

**Keywords:** Long-term survivors, non-small cell lung cancer, surfactant proteins

## Abstract

**Objective:** Long-term survivors (LS) of non-small cell lung cancer (NSCLC) without driver alterations, displaying an overall survival (OS) of more than 3 years, comprise around 10% of cases in several series treated with chemotherapy. There are classical prognosis factors for these cases [stage, Eastern Cooperative Oncology Group (ECOG), etc.], but more data are required in the literature. In this multi-center study, we focused on LS of advanced NSCLC with OS above 36 months to perform a clinical-pathological and molecular characterization.

**Methods:** In the first step, we conducted a clinical-pathological characterization of the patients. Afterwards, we carried out a genetic analysis by comparing LS to a sample of short-term survivors (SS) (with an OS less than 9 months). We initially used whole-genome RNA-seq to identify differentiating profiles of LS and SS, and later confirmed these with reverse transcription-polymerase chain reaction (RT-PCR) for the rest of the samples.

**Results:** A total of 94 patients were included, who were mainly men, former smokers, having adenocarcinoma (AC)-type NSCLC with an ECOG of 0–1. We obtained an initial differential transcriptome expression, displaying 5 over- and 33 under-expressed genes involved in different pathways: namely, the secretin receptor, surfactant protein, trefoil factor 1 (TFF1), serpin, Ca-channels, and Toll-like receptor (TLRs) families. Finally, RT-PCR analysis of 40 (20 LS/20 SS) samples confirmed that four genes (surfactant proteins and SFTP) were significantly down-regulated in SS compared to LS by using an analysis of covariance (ANCOVA) model: *SFTPA1* (*P* = 0.023), *SFTPA2* (*P* = 0.027), *SFTPB* (*P* = 0.02), and *SFTPC* (*P* = 0.047).

**Conclusions:** We present a sequential genetic analysis of a sample of NSCLC LS with no driver alterations, obtaining a differential RNA-seq/RT-PCR profile showing an abnormal expression of SF genes.

## Introduction

Non-small cell lung cancer (NSCLC) is currently one of the cancers with the highest mortality rates worldwide. According to the literature, in the pre-immunotherapy era, the 5-year survival rate of this aggressive disease was only 16%. Indeed, metastatic NSCLC is currently an incurable disease, and both chemotherapy and immunotherapy have only a palliative role in its treatment^1^.

Chemotherapy for NSCLC is based on platinum for a first-line setting. A meta-analysis of 16 randomized controlled trials has demonstrated that platinum-based chemotherapy may have a better potential to prolong survival than the best supportive care for these patients^2^. However, the prognosis for patients with advanced NSCLC is still poor. The median survival of these patients is around 12 months, while the 1-year survival rate is just 30%–35%, with 4%–6% of them being long-term NSCLC survivors (LS) displaying a survival time of more than 2 years in several series^3^.

Epidermal growth factor receptor (EGFR) mutations (in 15%–17% of patients), anaplastic lymphoma kinase-echinoderm microtubule-associated protein-like (ALK-EML) (2%–5%) and *ROS-1* (1%) translocation have been recently described in NSCLC patients. Target-specific treatments with tyrosine kinase inhibitors (TKIs) for these molecular alterations have demonstrated a significant benefit in both progression-free survival (PFS) and overall survival (OS), as well as a higher proportion of LS compared to chemotherapy^4–6^.

Recent developments in NSCLC diagnosis through next generation sequencing (NGS) techniques have allowed researchers to obtain more information about other potential treatable alterations (e.g., *HER2*, *RET*, *MET*, *BRAF*, and *NTRK*) that present in very low frequencies (1%–2%). In spite of their low prevalence, their identification is important because it allows treatment with certain targeted therapies that have a promising efficacy^7^.

A deeper analysis of LS patients without driver mutations that have undergone sequential chemotherapy treatments has rarely been explored in the literature. Why a small group of our patients without identified specific molecular alterations was able to reach an OS longer than 36 months is an unanswered question. Unknown molecular profiles involved in the response to chemotherapy, the tumor prognosis, or the impact of sequential local and systemic treatments could be some of the explanations. Previous studies about this group of patients without driver alterations include small numbers of patients and are focused on their clinical features alone, or alternatively on specific molecular findings. An integrative clinical–pathological and molecular analysis, as we propose in this research, has not been previously described.

Thus, in this study, we aimed for a complete characterization of advanced NSCLC LS displaying EGFR wild-type (wt) and non-translocated (nt) ALK/ROS1. We performed a thorough clinical and pathological treatment, and molecular characterization of a sample of advanced NSCLC patients from different institutions in Madrid who survived more than 36 months.

The primary objective of this study was to describe the basic clinical, pathological, and therapeutic characteristics in all the patients. In addition, we also analyzed both the OS and the PFS of all LSs included in the study.

A co-primary objective in this study was a molecular analysis of all the patients included. For this purpose, a sequential strategy of NGS (RNA-seq) and RT-PCR was used in order to identify specific molecular profiles of gene expression for these patients.

## Materials and methods

### Patients and samples

We retrospectively analyzed data from 94 patients at nine institutions in Madrid, Spain, who had been diagnosed with advanced NSCLC and had an EGFR wt/ALK-ROS1 nt genotype and an OS of at least 36 months. All these patients were selected from within a period of 14 years (from January 2002 to December 2016). We analyzed the clinical–pathological characteristics (age, sex, stage IIIB *vs.* IV, histology, smoking status, diabetes and cardiovascular disease, weight loss > 10%, symptoms at diagnosis, and sites of metastasis). Performance status (PS) was categorized from 0 to 4, according to the scale of the Eastern Cooperative Oncology Group (ECOG). Laboratory parameters [lactate dehydrogenase (LDH) and hemoglobin levels] and type of treatment administered (platinum-based treatment, metasectomies, chemotherapy lines, maintenance, grade 4 toxicity, and metformin intake) were also analyzed. Finally, PFS and OS data were also collected (**Table 1**).

For the molecular analysis, a control sample of 25 patients, including short-term survivors (SS) defined as having an OS less than 9 months, was also included. Histology, gender, PFS, and OS were among the clinical data collected from the SS patients.

This study was approved by our Ethics Committee, and all the patients signed an informed consent form during the recruitment process.

### Molecular analysis

**Figure 1:**
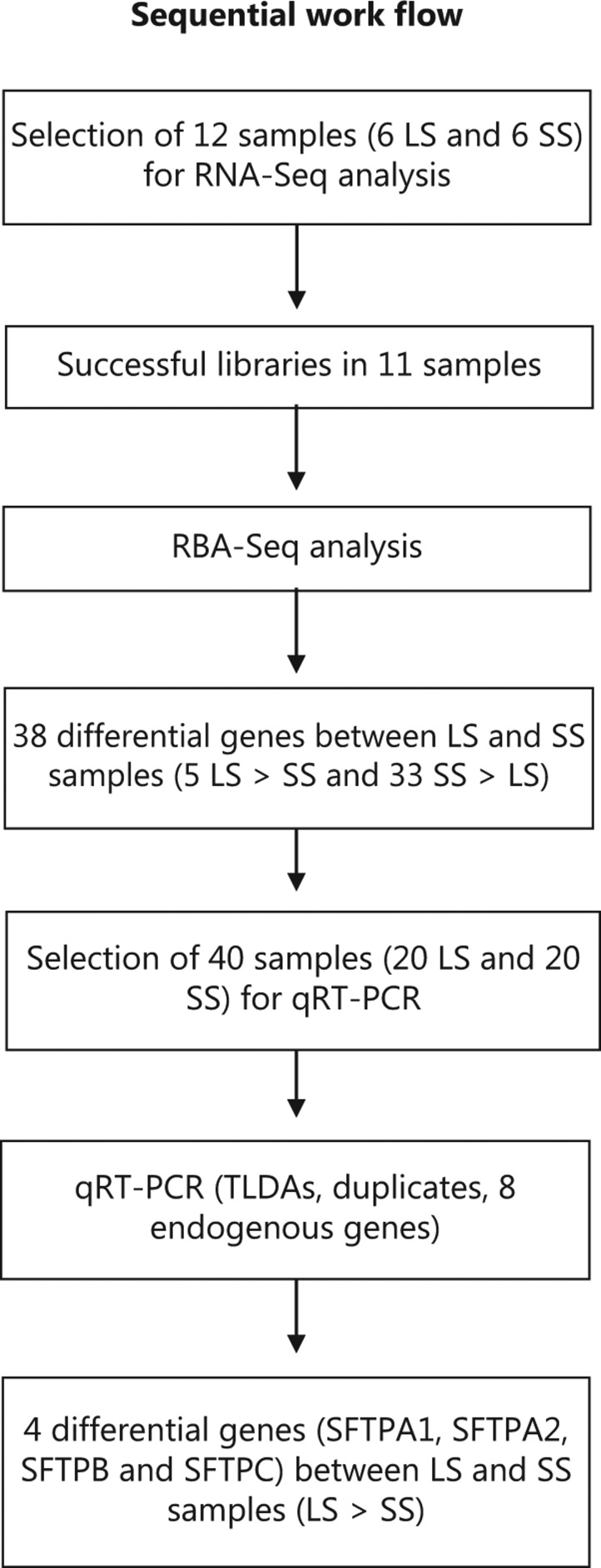
Sequential molecular analysis: indirect screening through RNA-Seq and further directed screening through reverse transcription-polymerase chain reaction (RT-PCR).

Molecular analysis was carried out with a sequential process comprising an initial untargeted whole-genome screen for differentially-expressed genes through RNA-seq technology, followed by a targeted screen with quantitative (q)RT-PCR using the genetic profile previously obtained (**Figure 1**).

Untargeted screening of candidate genes: for the initial RNA-seq experiment, 11 samples were selected, namely six samples from LS patients and five samples from SS. Successful libraries were obtained in all the 11 samples.

Targeted screening: For the quantitative PCR study (qRT-PCR), 114 samples were collected were collected, but we were not able to use most of the samples due to their poor quality or the lack of associated clinical data. We therefore performed the qPCR study with a subset of 40 samples (20 from LS patients and 20 from SS patients).

Institutional approval from our Ethics Committee was obtained for conducting the study. The ethical aspects are in accordance with Resolution CNS 196/96 and its complementary resolution. The samples were analyzed by two independent pathologists.

### Construction of RNA-seq libraries and analysis

To identify the differentially expressed genes between the tumors of the LS and SS patients, a previous exploratory study using the RNA-seq technology was carried out. RNA sequencing (RNA-seq), also called whole transcriptome shotgun sequencing (WTSS), is a developed approach to transcriptome profiling that uses NGS. RNA-seq can reveal the presence and quantity of RNA in a biological sample at a given moment. RNA-seq provides a far more precise measurement of the levels of transcripts and their isoforms than other methods.

RNA-seq libraries were generated from tumor samples embedded in paraffin. Previously, by means of a hematoxylin and eosin (H&E) staining, it was determined that the samples were composed of at least 80% tumor cells. These libraries were prepared according to the instructions of the NEBNext Ultra Directional RNA Library Prep kit for Illumina (Illumina, San Diego, CA, USA), following the protocol of the Poly(A) mRNA Magnetic Isolation Module to focus sequencing towards the 3′ end of transcripts. The input yield of total RNA to start the protocol was around 1 μg, as estimated by the Agilent 2100 Bioanalyzer using an RNA 6000 Nano LabChip kit (Agilent Technologies, Santa Clara, CA, USA). RNA integrity number (RIN) values were low, consistent with the characteristics of formalin-fixed paraffin-embedded (FFPE) samples, and so RNA fragmentation time was reduced accordingly. The library amplification phase included within the citied protocol was performed by applying a PCR of 18 cycles. The libraries obtained by these means were validated and quantified using an Agilent 2100 Bioanalyzer and a DNA 7500 LabChip kit (Agilent Technologies, Santa Clara, CA, USA). An equimolecular pool of libraries was prepared, cleaned using AmpureBeads (Beckman) and finally titrated by qPCR using the Kapa-SYBR FAST qPCR kit for LightCycler480 (Kapa Biosystems Inc., Wilmington, MA, USA) and a reference standard for quantification (Parque Científico de Madrid, Madrid, Spain). The pool of libraries was denatured prior to being seeded on a NextSeq flow cell at a density of 2.2 pM for clustering. Samples were then sequenced using a NextSeq™ 500 High Output Kit (Illumina, San Diego, CA, USA), in a format of 1 × 75 single read sequencing. An average of 41 ± 14 millions of pass-filter reads was obtained for the group of 11 samples, which were used for further filtering and bioinformatics analysis. Sequences were processed to remove possible artefacts and were then aligned against the human genome using the Bowtie aligner.

### RNA extraction and qPCR

The sections of FFPE tumors from the samples of the 119 patients (94 LS + 25 SS) were reviewed by two expert pathologists, as mentioned previously. A total of 40 samples were randomly selected after excluding cases with insufficient tumor cells or incomplete clinical data. More than 80% enrichment of the tumor cells was ensured, when necessary, by subsequent macro dissection with the use of a safety blade and a new confirmatory staining by H&E. The RNA was extracted from 10 to 15.5 μm sections using the AllPrep DNA/RNA FFPE Kit, an RNA purification kit (Qiagen, Hilden, Germany), according to the manufacturer’s instructions. Then qRT-PCR was performed using the LightCycler® 480 sequence detection system (Roche Diagnostics, Penzberg, Germany) according to the manufacturer’s instructions. After its design, the TaqMan Low Density Arrays (TLDA) were manufactured and supplied by AnyGenes (AnyGenes, Paris, France).

### Statistical analysis

Survival was recorded from the first day of treatment to the date of death or last follow-up, and the survival curves were calculated according to the Kaplan–Meier method. Description of qualitative data was made by using absolute frequencies and percentages and quantitative data by mean and standard deviation (SD).

In the untargeted screening of genes through RNA-seq statistical analysis, genes were assigned and differential expression [as fragments per kilobase of transcript per million mapped reads (FPKM) values] between LS and SS patients was determined by using EdgeR (Suite G-PRO, Biotechvana, Valencia, Spain).

In the case of the targeted screening of genes by qRT-PCR, analysis of covariance (ANCOVA) models were used to test the association of gene expression with survival group, using sex, age, histology, and stage as adjustment variables. Gene expressions were normalized by subtracting the average gene expression of three control genes: *GAPDH*, *ACTB* and *TBP*. A total of 41 genes were thereby tested, and the *P*-value of the gene expression variable was adjusted for multiple tests using the Bonferroni method to avoid inflation of Type I error. A significance level of 0.05 was used with two-tailed tests. The R 3.5.1 software was used throughout all the calculations.

## Results

### Clinical-pathological data

**Table 1** shows all the data collected concerning the clinical-pathological analysis for all 94 patients. All of the patients (hereafter pts) selected had been previously diagnosed with NSCLC with an OS of at least 36 months (m). All of them had been diagnosed and treated at one of nine institutions in Madrid: Infanta Sofía University Hospital (8/94 pts, 8.5%); Ramón y Cajal University Hospital (13/94 pts, 14.8%); San Carlos University Hospital (11 pts, 12.7%), Gregorio Marañón University Hospital (17 pts, 19.1%), 12 de Octubre University Hospital (30 pts, 32.9%); Alcorcón Foundation Hospital (8 pts, 8.5%), Príncipe de Asturias University Hospital (3 pts, 3.1%), La Paz University Hospital (2 pts) and Torrejón University Hospital (2 pts).

Regarding age, of the 94 patients, 55 (58.5%) were 65 years or older. Sixty-seven patients (71%) were male and 84 out of 94 (89.4%) had smoked or were current smokers. Comorbidities (diabetes and vascular disease) were infrequent: 7 pts (7%) and 13 pts (14%), respectively. Concerning performance status, we found ECOG 0 in 32 pts. (34%) and 1 in 57 pts (61%). Only 12 pts (14%) had weight loss greater than 10% at presentation. Cough (41 pts, 44%), followed by pain (34 pts, 36%), dyspnea (25 pts, 27%) and hemoptysis (9 pts, 9%) were the main symptoms, and the majority of LDH as well as hemoglobin levels were normal in the majority of patients.

Adenocarcinoma (AC) was the most common pathological subgroup (60 pts, 65%), followed by squamous (21 pts, 22%), large cell carcinoma (7 pts, 7%) and “other subtypes” (6 pts, 6%). One or two metastatic sites at diagnosis were described in 44 pts (47%) and 29 pts (31%), respectively, and only 13 pts (14%) had brain metastasis and 5 pts (5%) had adrenal metastasis.

First-line chemotherapy based on platinum was administrated in 92 pts (98%), and maintenance was used only in 41 pts (44%). Local treatment (metasectomies and/or radiation), was done in 35 pts (38%). Grade 4 toxicity was detected in 7 pts (7%).

**Figure 2:**
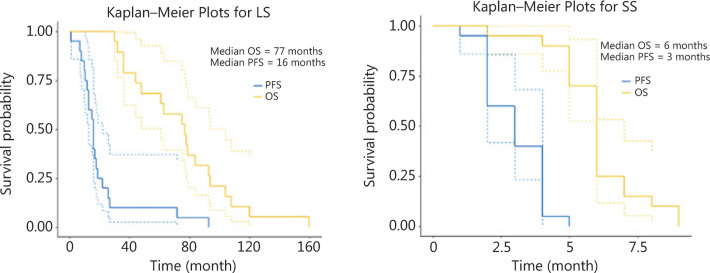
Progression free survival (PFS) and overall survival (OS) Kaplan–Meyer curve from advanced non-small cell lung cancer (NSCLC) long survivors (LS) and short survivors (SS) participating in the study.

Finally, a descriptive survival analysis was performed. The medians of PFS and OS were 16 months (14 m–32 m) and 77 months (36 m–80 m), respectively (**Figure 2**).

Among cases of SS included for the molecular comparative analysis, 14/26 were AC, 10 were squamous and 2 were large-cell carcinoma. Median PFS and OS were 3 months (1 m–4 m) and 6 months (4 m–9 m), respectively (**Figure 2**).

### Molecular analysis: potential involvement of surfactant proteins in long-term survival of NSCLC patients

**Figure 3:**
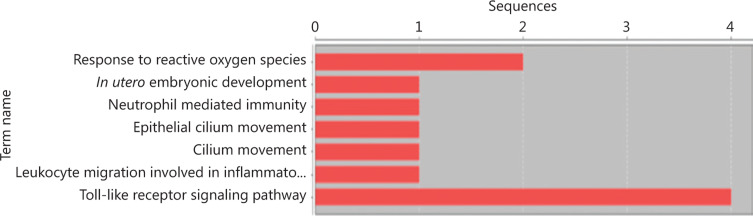
Histogram with number of sequences with GO annotations for ontology “Biological Process” in RNA-Seq analysis.

In this study, we have carried out a sequential molecular analysis (**Figure 1**). An initial NGS exploration was carried out on a limited and representative sample of six LS and five SS, according to sex, histology, and age, and a good quality of the sample embedded in the paraffin block. In this primary analysis, we obtained a significant differential transcriptome expression between samples from six LS and five SS, including five over-expressed genes in LS compared to SS and 33 over-expressed genes in SS compared to LS (**Table 2**). These genes were involved in different cellular pathways: the secretin receptor, surfactant protein, TFF1, the serpin family, the Ca-binding protein channel and the Toll-like receptor (TLRs) family (**Figure 3**).

**Figure 4:**
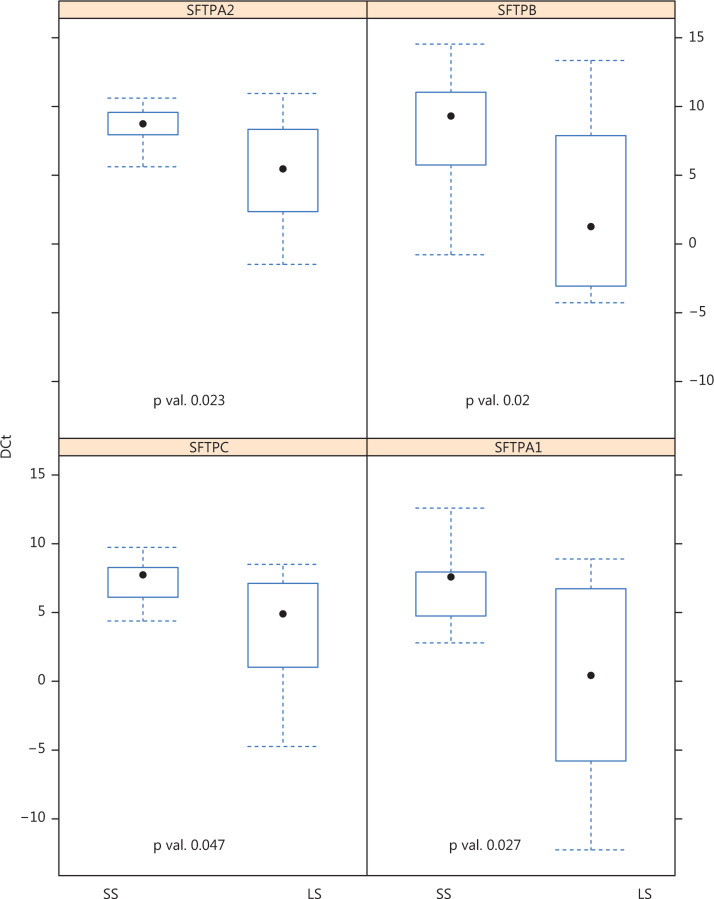
Genes obtained by reverse transcription-polymerase chain reaction RT-PCR with statistical significance resulting after RNA-Seq profile confirmation in two opposite samples with 20 long survivors (LS advanced lung cancer patients) and 20 short survivors (SS advanced lung cancer patients).

Finally, we conducted a confirmatory qRT-PCR of the previously-identified differentially expressed genes in 40 samples of both SS (20) and LS (20). By using ANCOVA models to analyze the data, it was determined that only four genes codifying for surfactant proteins were significantly down-regulated in SS compared to LS: *SFTPA1* (*P* = 0.023), *SFTPA2* (*P* = 0.027), *SFTPB* (*P* = 0.02), and *SFTPC* (*P* = 0.047) (**Figure 4**).

## Discussion

New treatment options are available for NSCLC, such as targeted therapy, immunotherapy, and chemo–immunotherapy combinations. However, the LS in advanced settings who were treated with chemotherapy previous to the immunotherapy era comprise a small subset of the NSCLC patients, characterized by a median OS of around 14–18 months. As we previously mentioned, the 5-year survival rates of these patients vary from 9% to 15%^2^.

In this retrospective study, we have included a significant sample of very LS of NSCLC without EGFR mutations or ALK/ROS1 translocations, and with a median OS of 36 months, at nine hospitals in Madrid. This is the sample of patients who lived the longest with these characteristics reported in the reviewed literature.

### Patient-related factors

In our study, 55/94 (58%) of the patients were 65 years or older, most were male (67/94, 71%) and the proportion of non-smokers was very low (9/94, 9.6%). However, regarding prognosis factors, females classically have a better prognosis; lung cancer incidence and prevalence have been described to be higher among men than women. In addition, EGFR, ALK and ROS1 alterations (excluded here) are more prevalent among women than men. Age is a controversial prognostic factor in lung cancer. Whereas different studies did not find a significant relation between age and long-term survival^8^, other authors consider older age to be a poor prognostic factor, mainly in those patients with potentially resectable disease^9^. This finding could be explained by comorbidities and tolerability to treatments, including surgery, in elderly patients compared to younger ones. However, in our sample, similar rates among patients both older and younger than 65 years were found, suggesting that it is probable that age is not a differential feature for becoming a LS in this setting.

As previously mentioned, the majority of patients included were current or former smokers, very similar to other series of lung cancer LS^10^.

In this study, most of the patients had neither diabetes (87/94, 92.5%) nor vascular diseases (81/94, 86%) indicating a very fit population with no serious comorbidities. Inal et al.^11^ found that diabetes is a poor prognosis factor in lung cancer in multivariate analysis. Given the low rate of diabetic patients in our sample, this would suggest that diabetes could play a negative role in a lung cancer patient’s outcome. Research concerning metformin and lung cancer is emerging. Different authors have concluded that metformin intake in diabetic patients diagnosed with NSCLC could improve their prognosis^12^. Studies of metformin intake in combination with targeted therapies such as anti-EGFR inhibitors or antiangiogenic drugs (e.g., bevacizumab) have shown a synergistic effect. In our study, most of our patients did not take metformin (74/94, 79%), but all of our diabetic patients took metformin as their main antidiabetic treatment (7/7). In addition, 11 patients were treated with metformin without a diagnosis of type-2 diabetes because of a pre-diabetic condition. The small number of patients in this subgroup precludes the analysis of the impact in survival of metformin intake.

PS is a classical prognosis factor in lung cancer, and several researchers have concluded that a poor PS is related to a poor prognosis^13^. In our sample, only 5 out of 94 patients (5.3%) had an ECOG of 2, whereas 60% of patients had ECOG 1 and 34% had ECOG 0, suggesting that a good initial PS could be a potentially favorable prognostic factor.

In addition, in our sample we have observed different symptoms at presentation: cough, pain, hemoptysis, and dyspnea. However, no studies have been reported correlating these factors to a poorer prognosis. Walter et al.^14^ described a group of NSCLC patients where hemoptysis was reported by 21.6% as an initial symptom associated with cancer, and chest/shoulder pain was the only initial symptom with a significantly shorter diagnostic interval for cancer compared with non-cancer diagnoses (*P* = 0.003). In our LS series, hemoptysis at diagnosis was very infrequent (9/94 patients, 9.5%) followed by dyspnea (26%), cough (56%), and pain (63%). These data are similar to most of the studies focused on collecting symptoms of lung cancer at presentation: cough is the most common manifestation, followed by weight loss, dyspnea, and chest pain^15^.

Regarding weight loss, several authors have reported that it is a clear factor in poor prognosis. Sahin et al.^16^ found that a low nutritional index was clearly related to shorter OS, and Crvenkova et al.^17^ found that weight loss > 10% was a poor and independently poor prognosis factor for long-term survival after therapy. Illa et al.^18^ reported that nutritional risk screening is a significant predictor of tumor response. Therefore, according to their data, early detection of malnutrition could be important for the prognosis of cancer patients as well as for planning effective supportive care. In our series, the majority of patients (85%) at diagnosis had not lost more than 10% of their basal weight. These findings were in accordance with those of previous authors who correlated a long survival with a good nutritional status. However, other recent studies failed to demonstrate this hypothesis. For example, in stage IV patients with lung ACs, authors found that pre-diagnosis weight loss had a negative impact on PFS, but no such effect was noticed on OS^19^.

### Tumour-related factors

Histology has been classically discussed as a prognosis factor. In this study, we have found that AC was the most common pathological subgroup (60 pts, 65%) followed by squamous (SC) (21 pts, 22%), and large cell carcinoma (LCC) (7 pts, 7%). In a large review with more than 20,000 patients of early lung cancer who underwent lobectomy, 872 were diagnosed with adenosquamous carcinoma (ASC), 7,888 with squamous cell (SC), and 12,601 with AC^20^. Shi found that survival after lobectomy for stage I and II disease was significantly reduced in ASC and SC compared to AC (*P* < 0.0001). ASC also had a significantly increased hazard ratio of 1.35 and 1.27 relative to AC and SC, respectively^21^. However, currently it is difficult to know if the pathology subtype itself involves a worse prognosis, or if molecular alterations associated with them could induce the outcome of these lung cancer patients. In our work, LS patients have an AC subtype, which is consistent with the literature conferring a better outcome^22^.

The number of metastasis sites has been studied as a potential feature associated with long-term survival. Other series of LS showed that a single metastasis location, as opposed to more than one location, was associated with a better outcome (62% *vs*. 41%, *P* = 0.008). In our sample, most of the patients have one or two metastasis sites (44 patients, 47%, and 29 patients, 31%, respectively), in agreement with the previous literature. The brain is a very common site of metastasis in patients with NSCLC, and we found only 13 patients (14%) with a single brain metastasis and 5 (5%) with adrenal metastasis who had undergone surgery^23,24^. Moscetti’s study found three cases (16.7%) of stage IV, 5-year treated with resection associated with improvement of long-term survival^25^.

### Treatment-related factors

Chemotherapy is the standard first treatment for NSCLC with ALK/ROS1 nt and EGFR wt genotypes. A platinum-based regime is the most common treatment in advanced lung cancer, associated with an OS of 10–14 months. Maintenance strategy has improved survival in those patients with response after induction^26^. First-line chemotherapy with a platinum-based regime was administered to 92 patients (98%); however, maintenance therapy was only administered to 41 patients (47%). No differences among platinum combinations have been demonstrated^27^, although different authors demonstrated a preferred combination such as AC and cisplatin-pemetrexed and squamous and cisplatin-gemcitabine^28^. In this series, only half of the patients (45 pts, 47%) received maintenance therapy, and therefore, we cannot affirm that this strategy is related to a better outcome.

Local treatments like metasectomies were performed in 35 patients (38%). The brain is a very common site of metastasis in patients with NSCLC. As we previously mentioned, only 13 patients (14%) had brain metastasis while 5 (5%) had adrenal metastasis and in all cases had undergone surgery. In accordance with this, Hsiung et al.^29^ confirmed these findings in another study with patients with resected brain metastasis, whole brain radiation or chemotherapy, who lived longer compared to those who did not receive such treatment.

Grade 4 toxicity was detected in only 7 patients (8%). Hardy et al.^30^ concluded that the administration of chemotherapy agents for NSCLC was associated to short- and long-term toxicities, and that they could be related to a worse survival. However, they did not find a relation between the intensity and the survival. There are no studies positing chemotherapy toxicity as a predictor of long-term survival, although, obviously, treatment is withdrawn or doses are reduced in patients with severe toxicity to chemotherapy, with a potential negative impact on their survival.

Finally, in this sample, we estimated a median PFS of 16 months and a median OS of 77 months, which is even better than other LS series, such as that of Skaug et al.^31^ with a median survival of 2.9 years (ranging from 2 to 5.1 years), 4.1% being still alive after 36 months and 0.41% reaching over 5 years of OS.

### Molecular analysis

We further enhanced the current study by including a molecular analysis in order to identify differentially expressed genes in our sample of LS. We performed a whole-genome RNA-seq analysis followed by confirmatory qRT-PCR.

As previously described, a molecular exploration was carried by including all the patients with an adequate sample for an NGS analysis. In the first step, we selected 11 patients (6 LS and 5 SS) displaying an extremely different OS (> 36 months *vs.* < 9 months, respectively). They were representative of the rest of the patients (balanced histology and centers and good-quality pathologist specimens). We selected RNA-seq for this initial approximation to do a massive differential expression analysis. A significant differential profile between LS and SS was obtained (**Figure 3** and **Table 2**), with 5 genes over-expressed in LS compared to SS and 33 over-expressed in SS compared to LS in transcriptome expression. Different families of relevant bronchial mucosae genes were involved in these differences. For example, secretin receptor genes were over-expressed in LS compared to SS samples, and its role in lung cancer has been described by some authors. Korner et al.^32^ found wild-type and splice-variant secretin receptors in bronchopulmonary carcinoid, suggesting that these genes may play a role in peritumoral lung pathophysiology. Other authors also have analyzed secretin receptors’ alterations in different tumors (gastrointestinal, lung, and pancreatic cancer), describing a relevant implication for cancer physiopathology^33,34^. Trefoil factor 1 (*TFF1*) was also one of the genes involved in this differential expression among LS and SS. Trefoil factor family (TFF) domain peptides are thought to be involved in epithelial restitution of mucosa. It has been hypothesized that TFFs are also expressed on mucosal surfaces of the respiratory tract with inflammation, as well as in some cases with AC. These are very important in lung mucosa definitions; thus, an association of TFFs with bacteria was found in several studies and may contribute to the anti-microbial mucociliary system^35^. This fact could drive a special interest in new immune treatments and their interaction with respiratory microbiota. An ongoing analysis by our group is currently analyzing these genes and their relation to immunotherapy.

Septins are a family of cytoskeleton-related proteins with 14 members that associate and interact with actin and tubulin. In our study, septin family genes are also detected with a potential role in these differences among LS and SS. Several authors have described an established relation between septin genes and tumorigenesis^35^. Liu et al.^36^ found that *SEPT2*, *SEPT8*, *SEPT9*, and *SEPT11* were consistently up-regulated and SEPT4 and SEPT10 were down-regulated in most cancer types investigated. Alterations in septin protein expression were found in many tumors, such as biliary cancer, hepatocarcinoma, ovarian, colorectal, and urological cancer; however, this is the first study describing a role of these genes in lung cancer^37^.

Regarding calcium-binding protein channels and lung cancer, some authors had associated dysregulations of genes codifying ion-channels with a cancer outcome. For example, in a genome-wide DNA methylation analysis, Bulk et al.^38^ identified the KCa 3.1 channel gene (*KCNN4*) promoter as being hypomethylated in an aggressive NSCLC cell line, suggesting the existence of more aggressive phenotype cells. Alterations in the Ca channel (activated chloride channel-2, *CLCA2*) were also detected in RT-PCR with circulating tumor cells (CTC) in patients with lung AC^39^. Therefore, according to our findings as well as those of others in the literature, cellular Ca^2^+ channels could be an important target in lung cancer treatment.

TLRs are a well-known family of pattern recognition receptors that play a key role in the host immune system. Some authors have found a correlation between rs4986791 polymorphism of a protein of this family (TLR4) and lung cancer^40^. Li et al.^41^ also concluded that TLR4 activation promoted the immune system evasion of lung AC.

In summary, the RNA-seq results of our first analysis are biologically consistent, and all the genes dysregulated in our samples have been previously described as having a potential role in carcinogenesis or cancer progression.

As we previously mentioned, we made a second confirmatory analysis with RT-PCR of the 55 genes over- and under-expressed in the RNA-seq analysis, included in the different families previously described: secretin receptor, surfactant protein, TFF1, serpin family, Ca-binding protein channel and TLR family. In this second analysis, 40 samples were used (20 LS and 20 SS). In this case, only several subtypes of genes codifying for surfactant proteins demonstrated a significant differential expression between the two groups in the ANCOVA models after correcting for multiple tests: *SFTPA1* (*P* = 0.023), *SFTPA2* (*P* = 0.027), *SFTPB* (*P* = 0.02), and *SFTPC* (*P* = 0.047) (**Figure 4**).

In a massive transcriptome analysis of normal tissue and lung cancer samples, the authors have defined a lung cancer-specific gene signature, containing *SFTPA1* and *SFTPA2* genes, which distinguished lung cancer from other cancer samples with a high predictive accuracy^42^. Additionally, Grageda et al.^43^ found in tumor and peri-tumor tissue that expression of *SFTPA2* mRNA and total SP-A protein was significantly lower in cancerous tissue compared to adjacent NC tissue (*P* < 0.001), suggesting that *SFTPA2* could be a biomarker for lung cancer diagnosis. Similar results were found by a Norwegian group while studying different markers in regional nodes and peripheral blood. They found *SFTPA* and *SFTPC* mRNAs to be potential markers, emphasizing a potential prognosis role of these proteins^44^. Some authors have demonstrated the over-expression of Pro-SFTPB in lung AC, but a prognosis value of these proteins has not been demonstrated^45^. Serum screening for lung cancer risk with Pro-SFTPB has been analyzed, indicating a potential value for lung cancer prediction^46^. Sin et al.^47^ also analyzed the role of SFTPC in stem cells in lung AC, and they found cancer stem cells in 52 out 57 samples; additionally, those with a bronchioalveolar phenotype SFTPC (+) were more differentiated and patients had a better prognosis compared to stem cells, which were related to a poorer prognosis. In our study, no alterations in SFTD were found, but a Japanese group found that SFTPD dysregulation could play a role in lung cancer^48^.

These surfactant proteins have been widely related to lung cancer identification in the literature However, less information about their role in cancer behavior has been described^49^. This is the first analysis demonstrating a correlation between lung cancer long-term survival and surfactant protein expression.

Therefore, among the strengths of this study we can highlight the following: the long follow-up period, the multi-institutional involvement and the relatively high number of long-term survival patients with advanced NSCLC included for clinical-pathological analysis. On the other hand, we must admit that we found a considerable decrease of valid cases for the molecular analysis because of tissue limitations. A further validation of this profile in a larger sample of patients is ongoing, including those LS treated with immunotherapy, to determine whether these results could also be applicable to a wider population. The role of these surfactant proteins in the bronchial immune system is widely known. A further validation of these profiles in lung cancer LS in the immunotherapy era is ongoing, primarily considering the potential role of surfactant proteins in immuno-regulation.

## Conclusions

This is the largest multi-center study reported involving very LS (OS > 36 months) without EGFR and ALK/ROS1 alterations in an exhaustive clinical and pathological analysis. The majority of these patients were male smokers or former smokers with AC and ECOG 0–1. An additional comprehensive molecular analysis found a specific and differential molecular profile for this sample.

## Figures and Tables

**Table 1:** Clinical, pathological and treatment collected data from all the LS patients included

Clinical-pathological characteristics	*n* = 94 pts	%
Age, ≥ 65/< 65 years	55/39	58.5/41.4
Gender, M/F	67/27	71.2/27.7
Smoking status, yes/no	85/9	90.4/9.6
Diabetes mellitus, yes/no	7/87	7.4/92.5
Vascular disease, yes/no	13/81	13.8/86.1
Stage, IV/IIIB pleural effusion	83/11	88.3/11.7
Pathology, LC/adeno/SQ/others	7/60/21/6	7.4/63.8/22.3/6.3
ECOG, 0/1/≥ 2	32/57/5	34/60.6/5.3
Cough at diagnosis, yes/no	53/41	56.3/43.6
Pain at diagnosis, yes/no	60/34	63.8/36.1
Hemoptysis at diagnosis, yes/no	9/85	90.4/9.5
Dyspnea, yes/no	25/69	26.6/73.4
> 10% Weigh loss, yes/no	14/80	14.8/85.1
Metastasis number location, 0/1/2/> 3	7/45/25/16	7.5/48.3/2 6.8/17.2
Brain metastasis, yes/no	13/80	13.9/86
Adrenal metastasis, yes/no	5/88	5.3/94.6
Metastasis surgery, yes/no	35/59	37.2/62.7
Platinum based regimen, yes/no	92/2	97.8/2.1
Maintenance, yes/no	45/49	47.8/52.1
- 2^nd^ Line, yes/no	72/22	23.4/76.5
- 3^rd^ Line, yes/no	53/41	56.3/43.6
- 4^th^ Line, yes/no	38/55	40.8/59.1
- 5^th^ Line, yes/no	26/67	27.9/72
Grade IV toxicity, yes/no	7/86	7.5/92.4
LDH levels > 1.5UNL//< 1.5ULN	29/65	30.8/69.1
Metformine intake, yes/no	16/70	18.6/81.4
Hemoglobin levels, ≥ 12/< 12	22/72	23.4/76.6

ECOG, Eastern Cooperative Oncology Group; M/F, male/female; LDH, lactate dehydrogenase; LC, lung cancer; LS, long-term survivor SQ, squamous.

**Table 2:** RNA-Seq analysis: Significant overexpression in LS compared to SS samples and CS compared to LS

Expression	*P*	Gene name	Gene description	Chromosome name	HGNC symbol
LS >> CS	2.93E-06	*SCTR*	Secretin receptor	2	SCTR
LS >> CS	1.99E-08	*SFTPA1*	Surfactant protein A1	10	SFTPA1
LS >> CS	2.37E-08	*SFTPA2*	Surfactant protein A2	10	SFTPA2
LS >> CS	1.03E-06	*SFTPB*	Surfactant protein B	2	SFTPB
LS >> CS	6.09E-10	*SFTPC*	Surfactant protein C	8	SFTPC
CS >> LS	2.11E-05	*AKR1B*10	Aldo-keto reductase family 1 member B10	7	AKR1B10
CS >> LS	1.92E-05	*C10orf99*	Chromosome 10 open reading frame 99	10	C10orf99
CS >> LS	2.10E-05	*C9orf84*	Chromosome 9 open reading frame 84	9	C9orf84
CS >> LS	2.00E-05	*CAPSL*	Calcyphosine like	5	CAPSL
CS >> LS	2.25E-05	*CXCL6*	C-X-C motif chemokine ligand 6	4	CXCL6
CS >> LS	4.22E-06	*DBT*	Dihydrolipoamide branched chain transacylase E2	1	DBT
CS >> LS	1.70E-05	*DNAI1*	Dynein axonemal intermediate chain 1	9	DNAI1
CS >> LS	1.37E-05	*DNAJC19*	DnaJ heat shock protein family (Hsp40) member C19	3	DNAJC19
CS >> LS	6.96E-06	FAM3D	Family with sequence similarity 3 member D	3	FAM3D
CS >> LS	1.18E-05	*FAM83B*	Family with sequence similarity 83 member B	6	FAM83B
CS >> LS	1.55E-05	*FSTL1*	Follistatin like 1	3	FSTL1
CS >> LS	1.78E-09	*GJB3*	Gap junction protein beta 3	1	GJB3
CS >> LS	1.45E-05	*KIF20A*	Kinesin family member 20A	5	KIF20A
CS >> LS	8.19E-06	*KRT14*	Keratin 14	17	KRT14
CS >> LS	6.18E-06	*KRT16*	Keratin 16	17	KRT16
CS >> LS	9.35E-07	*KRT17*	Keratin 17	17	KRT17
CS >> LS	8.98E-06	*KRT5*	Keratin 5	12	KRT5
CS >> LS	1.48E-05	*LCE3D*	Late cornified envelope 3D	1	LCE3D
CS >> LS	2.50E-06	*PDS5B*	PDS5 cohesin associated factor B	13	PDS5B
CS >> LS	1.70E-08	*PPBP*	Pro-platelet basic protein	4	PPBP
CS >> LS	9.44E-09	*S100A7*	S100 calcium binding protein A7	1	S100A7
CS >> LS	7.25E-06	*SAA2*	Serum amyloid A2	11	SAA2
CS >> LS	8.20E-06	*SAA2-SAA4*	SAA2-SAA4	11	SAA2-SAA4
CS >> LS	4.59E-06	*SAA4*	Serum amyloid A4, constitutive	11	SAA4
CS >> LS	7.54E-07	*SBSN*	Suprabasin	19	SBSN
CS >> LS	1.32E-08	*SERPINB4*	Serpin family B member 4	18	SERPINB4
CS >> LS	1.22E-05	*SPRR2B*	Small proline rich protein 2B	1	SPRR2B
CS >> LS	8.37E-06	*SPRR2F*	Small proline rich protein 2F	1	SPRR2F
CS >> LS	1.23E-05	*SPRR2G*	Small proline rich protein 2G	1	SPRR2G
CS >> LS	2.10E-07	*STYK1*	Serine/threonine/tyrosine kinase 1	12	STYK1
CS >> LS	2.21E-05	*TFF1*	Trefoil factor 1	21	TFF1
CS >> LS	1.36E-06	*TGM3*	Transglutaminase	20	TGM3
CS >> LS	2.03E-05	*UBE4B*	Ubiquitination factor E4B	1	UBE4B

CS, short survivors; LS, long-term survivors; SS; short-term survivors.
